# Endothelial cell and T‐cell crosstalk: Targeting metabolism as a therapeutic approach in chronic inflammation

**DOI:** 10.1111/bph.15002

**Published:** 2020-03-09

**Authors:** Michelangelo Certo, Hagar Elkafrawy, Valentina Pucino, Danilo Cucchi, Kenneth C.P. Cheung, Claudio Mauro

**Affiliations:** ^1^ Institute of Inflammation and Ageing, College of Medical and Dental Sciences University of Birmingham Birmingham UK; ^2^ Medical Biochemistry and Molecular Biology Department, Faculty of Medicine Alexandria University Alexandria Egypt; ^3^ Barts Cancer Institute Queen Mary University of London London UK; ^4^ School of Life Sciences The Chinese University of Hong Kong Hong Kong SAR China; ^5^ Institute of Cardiovascular Sciences, College of Medical and Dental Sciences University of Birmingham Birmingham UK; ^6^ Institute of Metabolism and Systems Research, College of Medical and Dental Sciences University of Birmingham Birmingham UK

## Abstract

The role of metabolic reprogramming in the coordination of the immune response has gained increasing consideration in recent years. Indeed, it has become clear that changes in the metabolic status of immune cells can alter their functional properties. During inflammation, T cells need to generate sufficient energy and biomolecules to support growth, proliferation, and effector functions. Therefore, T cells need to rearrange their metabolism to meet these demands. A similar metabolic reprogramming has been described in endothelial cells, which have the ability to interact with and modulate the function of immune cells. In this overview, we will discuss recent insights in the complex crosstalk between endothelial cells and T cells as well as their metabolic reprogramming following activation. We highlight key components of this metabolic switch that can lead to the development of new therapeutics against chronic inflammatory disorders.

**LINKED ARTICLES:**

This article is part of a themed issue on Cellular metabolism and diseases. To view the other articles in this section visit http://onlinelibrary.wiley.com/doi/10.1111/bph.v178.10/issuetoc

**Abbreviations**
3PO: 3‐(3‐pyridinyl)‐1‐(4‐pyridinyl)‐2‐propen‐1‐one
ACC: acetyl‐CoA carboxylase
AICAR: 5‐aminoimidazole‐4‐carboxamide ribonucleoside
AMPK: AMP‐activated protein kinase
APC: antigen‐presenting cell
BCH: 2‐aminobicyclo‐(2,2,1)‐heptane‐2‐carboxylic acid
CPT1A: carnitine palmitoyl transferase
D3T: 3*H*‐1,2‐dithiole‐3‐thione
EC: endothelial cell
FADH: flavin adenine dinucleotide
FAO: fatty acid oxidation
FASN: fatty acid synthase
G6PD: glucose‐6‐phosphate dehydrogenase
GLS1: glutaminase
HIF‐1α: hypoxia‐inducible factor‐1α
ICAM‐1: intercellular adhesion molecule‐1
IDO: indoleamine 2, 3‐dioxygenase
LDH‐A: lactate dehydrogenase A
LECs: lymphatic endothelial cells
LN: lymph node
MHC: major histocompatibility complex
OXPHOS: oxidative phosphorylation
PFK1: phosphofructokinase‐1
PFKFB3: 6‐phosphofructo‐2‐kinase/fructose‐2,6‐bisphosphatase 3
RA: rheumatoid arthritis
S1P: sphingosine‐ 1‐ phosphate
SLE: systemic lupus erythematosus
SREBP: sterol regulatory element‐binding protein
TCA: tricarboxylic acid cycle
TCR: T‐cell receptor
TGF: transforming growth factor
Th: T helper cell
VCAM‐1: vascular cell adhesion molecule

## 1 INTRODUCTION

Endothelial cell (EC)–T‐cell interactions play a key role in the regulation of the immune system. ECs are known to be of special importance for the control of T‐cell recruitment and activation. The endothelium represents an important interface between the tissues and the blood. Increasing evidence is highlighting how interaction between lymphocytes and ECs influences T‐cell activation and differentiation within the periphery, leading to ECs being considered as semi‐professional antigen‐presenting cells. Thus, T‐cell–EC interactions play a critical role in the regulation of the immune system during chronic inflammation. As ECs can be considered active participants in the processes of immune‐mediated inflammation, it is particularly important to have an in depth‐comparison between these two systems and to understand how they affect each other.

**FIGURE 1:**
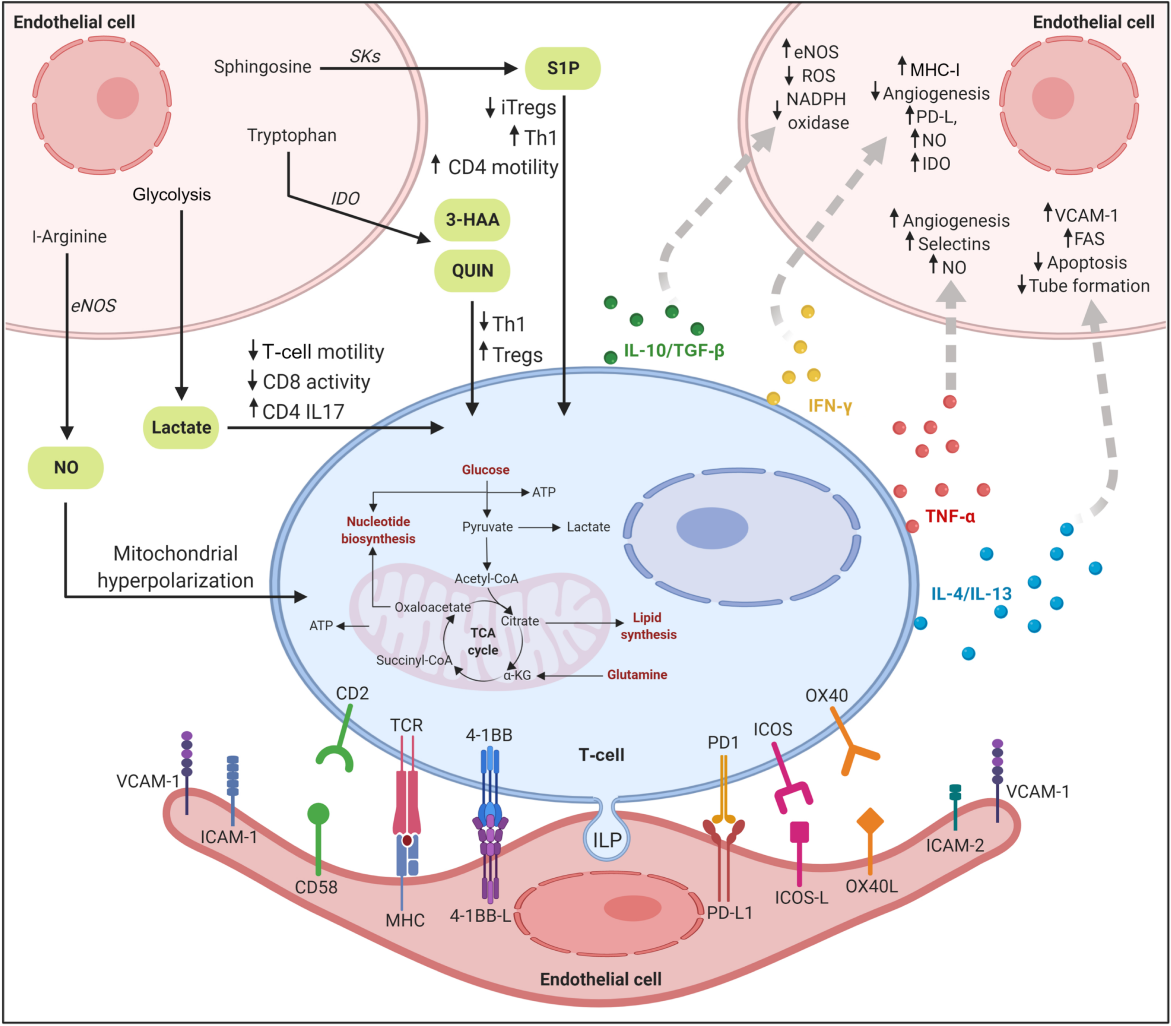
The T‐cell–endothelial cell immunological synapse. The lower part of the figure illustrates some of the membrane‐bound molecules that participate in antigen presentation and subsequent T‐cell activation by the endothelium. During lateral migration, T cells generate cylindrical structures, termed invadosome‐like protrusions, that enable close cell‐to‐cell contacts with endothelial cells and allow T cells to detect chemokines in the vicinity of the endothelial plasma membrane. The upper part of the figure illustrates some examples of how endothelial cells can modulate metabolic reprogramming of T cells through secretion of various immunomodulatory molecules, such as NO, lactate, tryptophan metabolites, and sphingosine‐1‐phosphate (S1P). The signals sent by T cells to modulate endothelial cell growth and function during inflammation are also illustrated. T cells can modulate the activity of endothelial cells through the release of specific cytokines such as the Th1 cytokines IFN‐γ and TNF‐α, the Th2 cytokines IL‐4 and IL‐13, and the Treg cytokines IL‐10 and TGF‐β. 3‐HAA, 3‐hydroxyanthranilic acid; FAS, fatty acid synthesis; QUIN, quinolinic acid; SKs, sphingosine kinases

ECs express class I and class II major histocompatibility complex (MHC)–peptide complexes on their surface and come into regular contact with circulating T cells. They can also process and present antigens (Hirosue et al., 2014) and they express both costimulatory and co‐inhibitory molecules, such as intercellular adhesion molecule‐1 (ICAM‐1), vascular cell adhesion molecule‐1 (VCAM‐1), ICOS‐L (CD275) and PD‐L1 (Figure 1). However, ECs do not express the co‐stimulatory molecules CD80 or CD86, and therefore, they cannot activate naïve T cells but only the effector/memory CD4 and CD8 lymphocytes (Perez, Henault, & Lichtman, 1998). Through the release of specific mediators, ECs can then promote the expansion of one T‐cell subtype over another. Activated T cells differentiate into three different known effectors—T helper (Th) cells (CD4 T cells), cytotoxic T cells (CD8 T cells), and regulatory T (Treg) cells, all of which have different functions in mediating immune responses and can provide both soluble and contact‐dependent signals to modulate and protect ECs.

Besides EC–T‐cell interactions, the complex interplay between their metabolic reprogramming plays a crucial role in the regulation of the immune response. Cellular metabolism is the sum of all the biochemical reactions that take place in a cell and is essential to generate components and energy used by cellular processes, such as synthesis, catabolism, and secretion of biomolecules. The demand for energy and substrates varies, depending on the state of activation or specific functions that the cell has to perform. This is particularly revealing for immune cells whose activation, proliferation, and engagement of effector functions are closely linked to changes in cellular metabolism. Furthermore, immune cells with different functions use several different metabolic pathways to generate energy and biosynthetic intermediates to support their specific functions, such as the production of distinct sets of cytokines. Thus, there is growing interest in understanding how metabolic pathways influence immune responses and, ultimately, disease progression. Similarly, there is evidence that ECs alter their metabolism to shift from quiescence to an active state and that EC metabolism is an important determinant of EC phenotype and behaviour (Tang & Mauro, 2017).

Understanding the mechanisms that drive metabolic reprogramming in ECs and T cells can lead to the explanation of the pathogenesis of numerous diseases. Hence, this review will analyse how activated ECs and T cells can reciprocally regulate their activities and how metabolic reprogramming of these cells during inflammation can be targeted to achieve the development of new therapeutics in chronic inflammatory diseases.

## 2 REGULATION OF T‐CELL ACTIVATION AND DIFFERENTIATION BY ECs

The crosstalk between ECs and T cells during the recruitment of T cells to sites of inflammation is strongly facilitated by the formation of a peculiar immunological synapse termed a “podo‐synapse.” Following initial antigen (Ag) recognition by T cells at the site of inflammation, the increased calcium influx induces formation of stabilized clusters of cylindrical actin‐rich invadosome‐like protrusions, which exert biomechanical and biochemical scanning of the endothelial surface and thus allow the lymphocytes to initiate diapedesis and to detect various chemokines on the endothelial plasma membranes (Martinelli et al., 2014; Figure 1). ECs express both class I and class II MHC molecules and a number of co‐stimulatory molecules whose receptors are present on activated T cells (Lim, Olding, Healy, & Millar, 2018). It has been suggested that human ECs might play a role in secondary immune responses by presenting antigen to circulating CD4 memory T cells (Pober & Tellides, 2012). In support of this, ECs can stimulate CD4 T cells in an antigen‐specific manner (Savage et al., 1995). However, human ECs do not express the co‐stimulatory ligands CD80 and CD86, and this can explain why they are not able to activate naive T cells. ECs also have a role in the recruitment of Treg cells (Tregs). Krupnick et al. (2005) have shown that alloantigen presentation by vascular endothelium to CD4 T lymphocytes activates and induces CD4^+^25^+^Foxp3^+^ Tregs, which further inhibit both in vitro and in vivo proliferation of alloreactive T cells. Another study has reported that, under inflammatory conditions, human ECs can induce proliferation of memory CD4 T cells, pro‐inflammatory Th17 cells, and Tregs (Taflin et al., 2011). Therefore, human ECs enhance T‐cell proliferation and increase Treg suppressor function.

Diseases with an important inflammatory component are often characterized by alterations of the endothelium, resulting in altered trafficking of immune cells. Several studies have reported a dramatically increased expression of endothelial MHC‐II in autoimmune/inflammatory diseases, such as diabetes, allograft rejection, multiple sclerosis, myocarditis, rheumatoid arthritis (RA), lupus, and vasculitis (Turesson, 2004). In these conditions, there is a change in the endothelial phenotype towards a pro‐inflammatory state, associated with increased expression of leukocyte adhesion molecules and cytokines, as well as reduced endothelium‐derived NO.

In this section, we will focus on how ECs can modulate metabolic reprogramming of T cells through the production of four immunomodulatory molecules, namely, NO, lactate, indoleamine 2,3‐dioxygenase (IDO), and sphingosine‐1‐phosphate (S1P), due to their significant direct involvement in the metabolic modulation of T cells. These are just a few examples, as there are different outcomes of specific EC–T‐cell interactions, strictly dependent on the cell phenotype and micro‐environment. Other important chemical mediators secreted by ECs and that can modulate T‐cell functions include different EC‐derived cytokines, such as IL‐6, IL‐11, IL‐12, and IL‐18, which are involved in the induction of T‐cell differentiation. Lymphatic ECs (LECs) can also secrete IL‐7, which is crucial for the regulation of naive and memory T‐cell homeostasis as well as for the access to secondary lymphoid organs. ECs of high endothelial venules, lining the blood vessels of lymph nodes (LNs), express certain chemokines, such as CCL19, CCL21, CXCL12, and CXCL13, that control lymphocyte trafficking to the LNs and Peyer's patches. These aspects have recently been reviewed in more details (Al‐Soudi, Kaaij, & Tas, 2017; Humbert, Hugues, & Dubrot, 2017).

### 2.1 Nitric oxide

NO is a diffusible transcellular messenger molecule, which is widely recognized as one of the key synthetic products of ECs because of its significant roles in many physiological and pathological conditions, including vasodilation and inflammation. NO generated by ECs is believed to affect the immune cells in a paracrine fashion as it can readily permeate cell membranes and modulate immune cell metabolism (Förstermann & Sessa, 2012; Figure 1). A study by Lukacs‐Kornek et al. demonstrated a bidirectional crosstalk between T cells and LECs. They reported that LECs inhibit T‐cell proliferation through a tightly regulated mechanism dependent on NOS2. In vitro production of NO by LECs was noticed upon activation of T cells and required the interplay of IFN‐γ and TNF and direct contact with activated T cells. Moreover, in vivo experiments showed that LECs in primed mice expressed NOS2 in the T‐cell zone. This was suggested as a mechanism to regulate the T‐cell proliferative effect and generation of NO‐induced Tregs (Lukacs‐Kornek et al., 2011). NO has been shown to stimulate glycolysis and to impair mitochondrial reserve capacity (Diers, Broniowska, Darley‐Usmar, & Hogg, 2011). Emerging evidence indicates that NO production might regulate mitochondrial biogenesis as it competes with molecular oxygen and reversibly inhibits cytochrome *c* oxidase, thus suggesting a possible role in the control of mitochondrial respiration (Nagy, Koncz, & Perl, 2003). NO triggered T‐cell apoptosis and played a significant role in Th1 cell‐mediated responses via IFN‐γ (Niedbala, Cai, & Liew, 2006). Furthermore, this molecule has nitrosylating effects on specific enzymes of glycolysis, tricarboxylic acid (TCA) cycle, and fatty acid (FA) metabolism. For example, Doulias, Tenopoulou, Greene, Raju, and Ischiropoulos (2013) have shown that endothelial NOS (eNOS)‐derived NO can induce *S*‐nitrosylation of very long acyl‐CoA dehydrogenase, which catalyses the first step in β‐oxidation, resulting in enhanced activation of its enzymatic activity. This could result in reduced expansion of Tregs, as these cells rely primarily on FA oxidation (FAO); at the same time, there could be a shift towards Th1, Th2, or Th17 subsets. NO has been shown to induce proliferation of functional Treg cell subsets, although the precise mechanism is currently unclear (Niedbala et al., 2006).

### 2.2 Immunosuppressive IDO

ECs can directly regulate immune responses via the expression of immunosuppressive IDO, which is the first and rate‐limiting enzyme in the catabolic pathway of the essential amino acid tryptophan. The enzyme is responsible for extra‐hepatic catabolic degradation of tryptophan, and it is expressed in vascular endothelium, professional antigen‐presenting cells, epithelial cells, and tumour cells (Gerriets & Rathmell, 2012). IDO is produced after ECs are triggered by IFN‐γ and/or TNF‐α (Mbongue et al., 2015) and is considered a major inhibitor of the immune response, as it appears to restrict potentially exaggerated inflammatory reactions (Wu, Gong, & Liu, 2018). Increased IDO expression and activity can increase the levels of bioactive metabolites that can control immune cell apoptosis and T‐cell polarization, thus shifting the balance between Th17 and anti‐inflammatory Foxp3^+^ Tregs (Terness et al., 2002; Figure 1). One mechanism by which IDO can modify the immune response is via tryptophan depletion in the local micro‐environment. In a very elegant study, Fallarino et al. (2002) showed that tryptophan metabolites in the kynurenine pathway, such as 3‐hydroxyanthranilic (3‐HAA) and quinolinic acids (QUIN), induce apoptosis of murine thymocytes and of Th1 but not Th2 cells in vitro. T‐cell apoptosis was associated with caspase‐8 activation and the mitochondrial release of cytochrome *c.* IDO‐mediated tryptophan insufficiency results in metabolic stress sensed by the stress‐responsive enzyme, general control non‐depressible (GCN2) kinase and the mechanistic target of rapamycin (mTOR), which eventually mediates T‐cell anergy and directs Treg transformation (Munn & Mellor, 2013).

### 2.3 Lactate

ECs rely on glycolysis as the main metabolic pathway for energy production (De Bock, Georgiadou, & Carmeliet, 2013) and hence excrete enormous amounts of lactate (Parra‐Bonilla, Alvarez, Al‐Mehdi, Alexeyev, & Stevens, 2010). Lactate is the final product of anaerobic glycolysis. It has been considered during most of the last century as a waste product, but new studies have attributed to lactate diverse metabolic and regulatory properties such as a source of metabolic energy (Boussouar & Benahmed, 2004), a modulator of energy production (Chari, Lam, Wang, & Lam, 2008), and a signalling molecule (Philp, Macdonald, & Watt, 2005). Interestingly, lactate modulates T‐cell function directly via specific cell surface lactate transporters. We have previously shown that sodium lactate is able to reduce the migration of CD4 T cells (leading to the entrapment of these cells in the inflamed tissue) by inhibition of glycolysis and to promote the expression of IL‐17 (Haas et al., 2015; Figure 1). Lactate is mainly produced in the cytoplasm during hypoxia or as a consequence of aerobic glycolysis in proliferating cells, and it is then secreted through the plasma membrane. This transport is dependent on solute carrier transporters that perform proton–lactate symport (MCT1‐4) or sodium‐dependent transport (SLC5A8 and SLC5A12). Lactic acid also enhances the production of IL‐23/IL‐17 by CD4 T cells, acting as a pro‐inflammatory signal (Yabu et al., 2011) and is a major fuel for the TCA cycle (Faubert et al., 2017; Hui et al., 2017). Sodium lactate selectively affects CD4 T‐cell functions via the solute carrier SLC5A12 (SMCT2), while lactic acid modulates CD8 T cells via its influx through SLC16A1, leading to the inhibition of their motility and cytotoxicity (Haas et al., 2015). We have recently reported that the increased IL‐17 production after lactate uptake by CD4 T cells depends on nuclear pyruvate kinase M2 (PKM2)/STAT3 and enhanced FA synthesis. Furthermore, lactate promotes CD4 T‐cell retention in the inflamed tissue via reduced glycolysis and enhanced FA synthesis (Pucino et al., 2019).

Lactic acid also exerts an inhibitory effect on cytotoxic activity of human and mouse NK cells, which is accompanied by down‐regulation of the expression of granzyme and perforins (Husain, Huang, Seth, & Sukhatme, 2013). In addition to its roles in metabolism, it has recently been proposed that lactate can stimulate gene transcription through epigenetic modifications. In particular, it has been shown that histone lactylation in M1 macrophages leads to the induction of the expression of M2 homeostatic genes, including ARG1, thus opening up new perspectives on the role of lactate in diverse pathophysiological conditions (Zhang et al., 2019).

In conclusion, lactate plays significant roles in the immunomodulation of T‐cell effector functions and can be considered a crucial messenger for EC–T‐cell crosstalk during inflammation.

### 2.4 Sphingosine‐1‐phosphate

S1P is a well‐known metabolite of LECs that regulate T‐cell egress from secondary lymphoid organs into the lymph and the blood. S1P is synthesized from sphingosine by sphingosine kinases 1 and 2. A recent study by Xiong et al. (2019) reported that S1P selectively enhances migration of human and murine CD4 T cell across LECs, promoting T‐cell egress from the LNs, in a process regulated by the S1P receptors 1 and 4 in CD4 T cell as well as S1P receptor 2 in ECs (Figure 1). The secretion of S1P involves two transporters, the nonspecific ATP‐binding cassette transporter and the recently identified specific transporter spinster 2 (Fukuhara et al., 2012).

In addition to its chemotactic function, recent evidence suggests that S1P can maintain naïve T‐cell survival. Mendoza et al. (2017) reported that S1P favours naive T‐cell survival by maintaining their mitochondrial content. In a study by Liu, Yang, Burns, Shrestha, and Chi (2010), it has been shown that the differentiation of pro‐inflammatory Th1 cells and Tregs is regulated by S1P1, which signals through mTOR and antagonizes TGF‐β function mostly by decreasing Smad3 activity. The study also proposed an S1P1–mTOR axis to control T‐cell lineage where two unrelated immunosuppressants, FTY720 and rapamycin, were used to target the same S1P1 and mTOR pathway to regulate the reciprocal differentiation of Th1 cells and Tregs. The S1P1 receptor has long been an established target of immune therapies aiming at sequestering T cells in the LNs. Such treatments, in the light of these studies, might have unexpected effects on T‐cell survival as well as sequestration. Further studies to reveal the underlying mechanism of S1P effects on immune cell metabolism, as well as the possible similar effects of other sphingolipids such as ceramides on T cells, are required.

In conclusion, the role of ECs in the regulation of the immune response is ever more appreciated. ECs contribute to the inflammatory process not only via angiogenesis and attraction of immune cells but also through the production of various immunomodulatory molecules with important immunoregulatory functions. In the last section of the review, we discuss approaches to target these processes in anti‐inflammatory therapies.

## 3 EC MODULATION BY T CELLS

Several studies have shown that T cells play significant roles as regulators of EC functions during inflammation through secretion of many immunomodulatory molecules and cytokines. Though CD4 Th cells are crucial for proper host defence and immune response, they are also major players in autoimmune conditions and inflammatory diseases. Cytokines are classified into pro‐inflammatory and anti‐inflammatory and are linked to Th subsets expressing them, such as Th1, Th2, Th17, Th22, and Th9 cells and Tregs, which are characterized by specific cytokine profiles. Among the cytokines produced by Tregs, IL‐10 and TGF‐β have anti‐inflammatory effects and play an important role in the modulation of the EC response. Following interaction with ECs, Foxp3^+^ iTregs can reduce leukocyte recruitment and EC activation. The protective role of IL‐10 is mainly due to its antioxidant properties, including inhibition of NADPH oxidase activity and ROS production (Gunnett, Heistad, & Faraci, 2002). TGF‐β can induce eNOS expression by activating Smad2, thus playing a protective role in maintaining EC homeostasis (Saura et al., 2002). These data also support the essential role of Tregs in immune suppression under physiological conditions. In this section, we will focus on selected Th1 cytokines (IFN‐γ and TNF) as well as Th2 cytokines (IL‐4 and IL‐13) as important chemical signals sent by T cells to modulate EC growth and functions during chronic inflammation.

### 3.1 IFN‐γ and TNF

IFN‐γ is a cytokine secreted by many immune cells, such as Th1 cells, NK cells, innate lymphoid cells, and cytotoxic CD8 T lymphocytes. Signalling by the IFN‐γ receptor activates the JAK–STAT1 pathway, which further up‐regulates the expression of classical IFN‐stimulated genes with significant immunomodulatory functions during inflammation (Villarino, Kanno, & O'Shea, 2017). Upon T‐cell activation, aerobic glycolysis enhances IFN‐γ production, and its secretion is believed to be one of the most important mechanisms through which T cells can control EC growth and survival (Jung, Zeng, & Horng, 2019; Peng et al., 2016; Figure 1).

TNF is produced by Th1, CD8, and innate immune cells and is implicated in inflammation, anti‐tumour responses, and immune system homeostasis. During an inflammatory immune response, TNF exerts numerous effects on ECs via induced gene expression for various integrins, adhesion molecules, and MMPs (Sainson et al., 2008; Figure 1).

In a study by Kataru et al. (2011), T cells exerted inhibitory effects on LN angiogenesis via the secretion of IFN‐γ. Another study has also shown that IFN‐γ can directly modulate LEC proliferation and survival in vitro (Shao & Liu, 2006). IFN‐γ and TNF have negative effects on EC growth in vitro; moreover, the anti‐angiogenic properties of IFN‐γ have been shown in vivo (Sun, Wang, Zhao, Zhang, & Chen, 2019). On the contrary, it has been shown that TNF can stimulate angiogenesis through indirect proangiogenic signals and also through stimulation of VEGF (Ligresti, Aplin, Zorzi, Morishita, & Nicosia, 2011). In ECs, TNF stimulates the production of S1P, which as described above, exerts its proangiogenic properties and inhibits apoptosis via interactions with the EC differentiation gene (Edg) receptor family (Ozaki, Hla, & Leem, 2003). TNF receptor activation also results in VEGF up‐regulation via activation of the NF‐κB pathway. Several studies have revealed that T cells can regulate the level of both class I and class II MHC expression on ECs. A study by Goes et al. (1995) showed that basal expression levels of class I MHC expression on ECs were significantly reduced in IFN‐γ or IFN‐γ receptor knockout (KO) mice. In vitro, ECs were shown to lose MHC class II and reduce class I expression, which was restored with IFN‐γ treatment (Resch, Fabritius, Ebner, Ritschl, & Kotsch, 2015). T cell‐derived TNF activates ECs and induces the expression of the selectins subfamily of surface adhesion molecules, which in turn mediate leukocyte adhesion (Pober & Sessa, 2007). Another study showed that IFN‐γ significantly enhances EC expression of programmed cell death‐1 ligands, namely, PD‐L1 and PD‐L2, which have been implicated in the interaction between ECs and Tregs, leading to activation of Treg function and induction of Treg subpopulations (Lim et al., 2018). Activated T cells, through IFN‐γ and TNF secretion, induce NOS2 up‐regulation and generation of NO by LECs and thus inhibit further T‐cell proliferation (García‐Ortiz & Serrador, 2018). IFN‐γ signalling and NOS2 expression were found to be essential for the immunosuppressive properties of LECs (Lukacs‐Kornek et al., 2011). Moreover, IFN‐γ stimulates cultured human LN‐derived LECs to secrete immunosuppressive IDO, which in turn impairs CD4 T‐cell proliferation (Nörder et al., 2012).

### 3.2 IL‐4 and IL‐13

IL‐4 and IL‐13 are multifunctional lymphokines secreted by CD4 T cells during a Th2 cell‐mediated inflammatory response. IL‐4 and IL‐13 bind to the common type II IL‐4 receptor expressed by ECs, which is a heterodimer consisting of IL‐4 receptor α and IL‐13 receptor α, and they mediate their actions via JAK–STAT signalling pathways (Symowski & Voehringer, 2019). Upon activation by IL‐4 and IL‐13, ECs respond by secreting various chemokines, such as eotaxin‐3/CCL26, which is a functional ligand for CCR3, and by expressing surface adhesion molecules including VCAM‐1, which in turn enhances the local recruitment of Th2 cells, eosinophils, and basophils and thus mediates the late‐phase response (Figure 1). Several studies have investigated the angiogenic properties of IL‐13 and IL‐4, and it has been reported that EC‐soluble VCAM‐1 is significantly induced in response to IL‐4 and IL‐13 treatment. Moreover, up‐regulation of VCAM1 expression by IL‐4 and IL‐13 was enhanced when combined with TNF stimulation (Fukushi, Ono, Morikawa, Iwamoto, & Kuwano, 2000).

Complement‐mediated EC injury results in cell retraction, loss of membrane permeability, and gap formation. Several studies have shown that IL‐4 and IL‐13 play crucial roles in protecting ECs against various injurious effects during inflammation. A study by Grehan et al. (2005) has found that ECs incubated with IL‐4 or IL‐13, but not with IL‐10 or IL‐11, were protected from killing by complement and apoptosis induced by TNF‐α plus cycloheximide. IL‐4 and IL‐13 stimulation leads to increased EC FA and phospholipid synthesis, as well as preservation of mitochondrial functions, which in turn have protective effects against injury induced by complement (Black et al., 2010). It has also been reported that Th2 cells and their cytokines IL‐4 and IL‐13 exert negative effects on the formation of lymphatic vessels through down‐regulation of essential transcription factors of LECs and inhibit tube formation (Shin et al., 2015).

Despite the complexity of the EC and T‐cell crosstalk, there are now considerable data supporting the conclusion that these interactions are bidirectional and that a better understanding of the underlying mechanisms can help us achieve the development of new therapeutic agents in chronic inflammatory diseases.

## 4 METABOLIC PATHWAYS IN EC AND T‐CELL ACTIVATION

Both intracellular and extracellular signals are necessary for the activation of metabolic pathways involved in the activation, proliferation, and function of immune cells. Immune cells with different functions activate different metabolic pathways to cope with increased energy demand. It is also known that ECs can alter their metabolism to fulfil the energy requirements for proliferation and angiogenesis. This metabolic switch from quiescent to highly proliferative state is induced by several cytokines and chemokines secreted by various cells during the inflammatory immune response. Here, we discuss some of the most important metabolic pathways that support EC and T‐cell activation.

### 4.1 Glycolysis

Glycolysis is a cytoplasmic pathway that starts with the uptake of glucose from the extracellular environment and then breaks down glucose into two three‐carbon compounds to generate energy. The final product of glycolysis is pyruvate in aerobic conditions and lactate in anaerobic conditions. Under aerobic conditions, pyruvate is transported into the mitochondria where it undergoes oxidation by the Krebs cycle to produce ATP.

**FIGURE 2:**
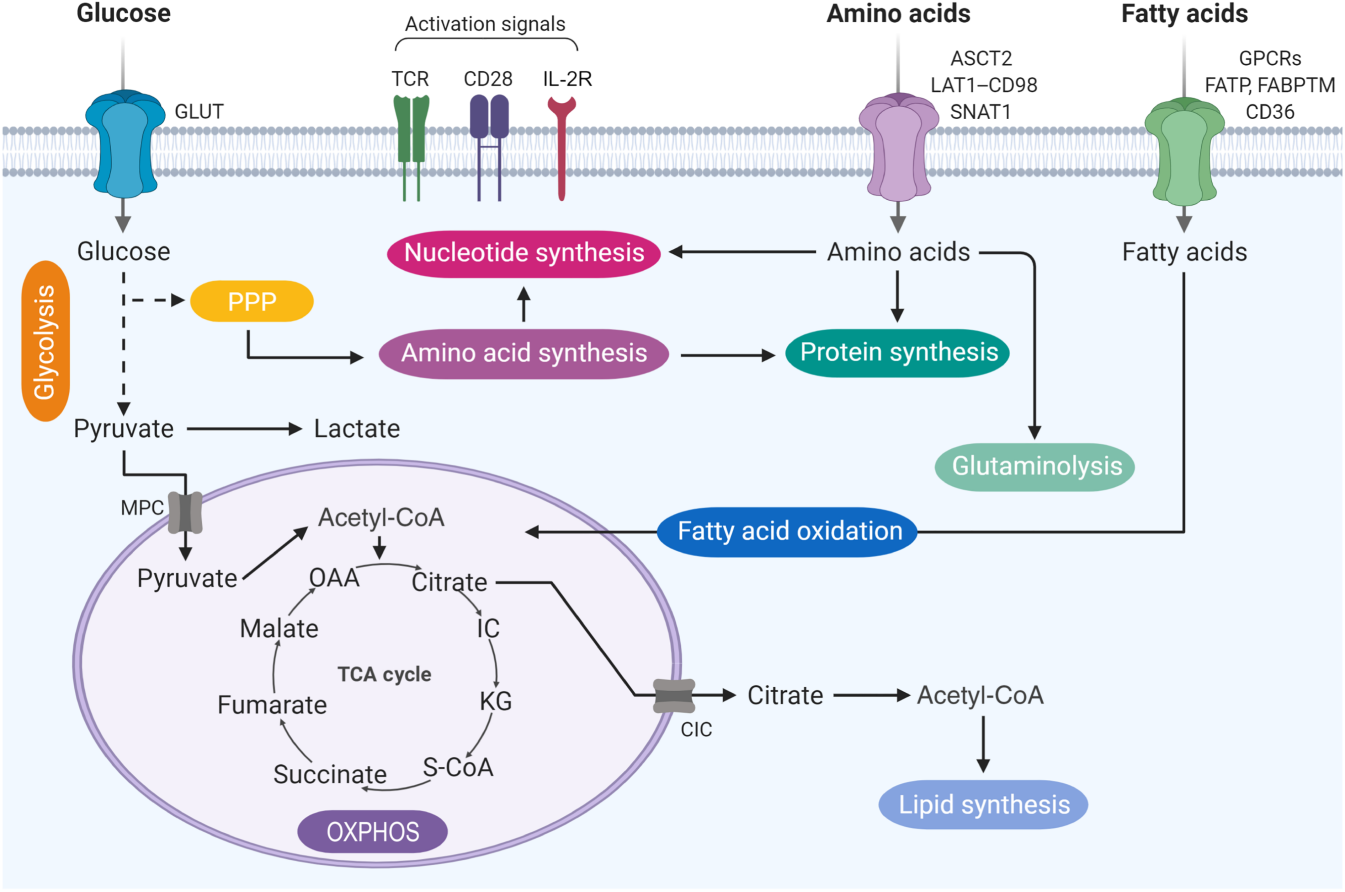
Metabolic pathways in activated T cells. After T‐cell receptor (TCR) engagement and CD28‐mediated co‐stimulation, activated T cells up‐regulate glucose transporters (GLUT) and amino acid transporters such as the glutamine transporter ASCT2, LAT1–CD98, which mediate leucine uptake, and the major alanine transporter SNAT1. These events allow T cells to undergo metabolic reprogramming that involves the up‐regulation of glycolysis with conversion of glucose into pyruvate. Pyruvate can either be converted to lactate or sustain the tricarboxylic acid (TCA) cycle. The citrate withdrawn from the TCA cycle can be used to sustain lipid synthesis. Glycolysis can also feed the pentose phosphate pathway (PPP), which produces the necessary intermediates for nucleotide and protein synthesis. Endogenous fatty acids or those imported from the extracellular space through T‐cell‐surface receptors, such as GPCRs, fatty acid‐binding proteins TM (FABPTM), fatty acid transport proteins (FATP), or CD36, can also be oxidized to sustain mitochondrial ATP production. CIC, citrate carrier; IC, isocitrate; IL‐2R, IL‐2 receptor; KG, ketoglutarate; MPC, mitochondrial pyruvate carrier; OAA, oxaloacetate; OXPHOS, oxidative phosphorylation; S‐CoA, succinyl CoA

Naïve T cells are metabolically quiescent, they need minimal nutrient uptake, and they couple the TCA cycle with oxidative phosphorylation (OXPHOS) for maximal ATP generation. By contrast, antigen‐activated T lymphocytes, like other proliferative cells (e.g., cancer cells), depend on aerobic glycolysis, a less efficient way to generate ATP (Vander Heiden, Cantley, & Thompson, 2009). This metabolic switch is known as the Warburg effect and refers to the observation that, even in the presence of oxygen, activated T cells tend to favour metabolism via glycolysis rather than the much more ATP‐efficient oxidative phosphorylation pathway. Indeed, glycolysis generates two molecules of ATP from one molecule of glucose, whereas OXPHOS can generate 36 molecules of ATP from one molecule of glucose. However, Warburg metabolism leads to increased availability of intermediates for the synthesis of nucleotides, amino acids, and FAs, which support cell division and proliferation (Figure 2).

In order to maintain aerobic glycolysis, T cells up‐regulate glucose transporters (GLUT; Mueckler & Thorens, 2013). GLUT1 expression and increased glucose influx are regulated by CD28‐dependent PI3K/Akt signalling (Frauwirth et al., 2002). In addition, murine T cells deficient in GLUT1 show altered proliferation and survival during activation (Macintyre et al., 2014). C‐myc is another important gene involved in the regulation of glycolysis and T‐cell development. Indeed, C‐myc KO mice die at the embryonic stage and C‐myc KO CD4 and CD8 T cells show impaired cellular proliferation and growth both in vitro and in vivo (Wang et al., 2011), and this is due to defective glycolysis and glucose influx (i.e., GLUT1 expression). Additionally, Myc regulates amino acid influx (i.e., Slc7a5 and Slc1a5 expression) and glutaminolysis suggesting its critical involvement in T‐cell activation‐dependent metabolic regulation (Wang et al., 2011).

mTOR and the hypoxia‐inducible factor‐1 (HIF‐1α) are transcription factors that act as important metabolic sensors. mTOR is critical in driving T‐cell differentiation and function (Waickman & Powell, 2012). HIF‐1α is activated during hypoxia and regulates glycolytic enzyme expression (Kaelin, 2005) and modulates differentiation and effector functions of CD4 and CD8 T cells (Doedens et al., 2013). In CD4 T cells, HIF‐1α regulates the balance between Th17 cells and Tregs (Pan, Barbi, & Pardoll, 2012). HIF‐1α KO CD4 T cells are defective in Th17 differentiation but more prone to differentiate into Tregs in vitro (Dang et al., 1999).

**FIGURE 3:**
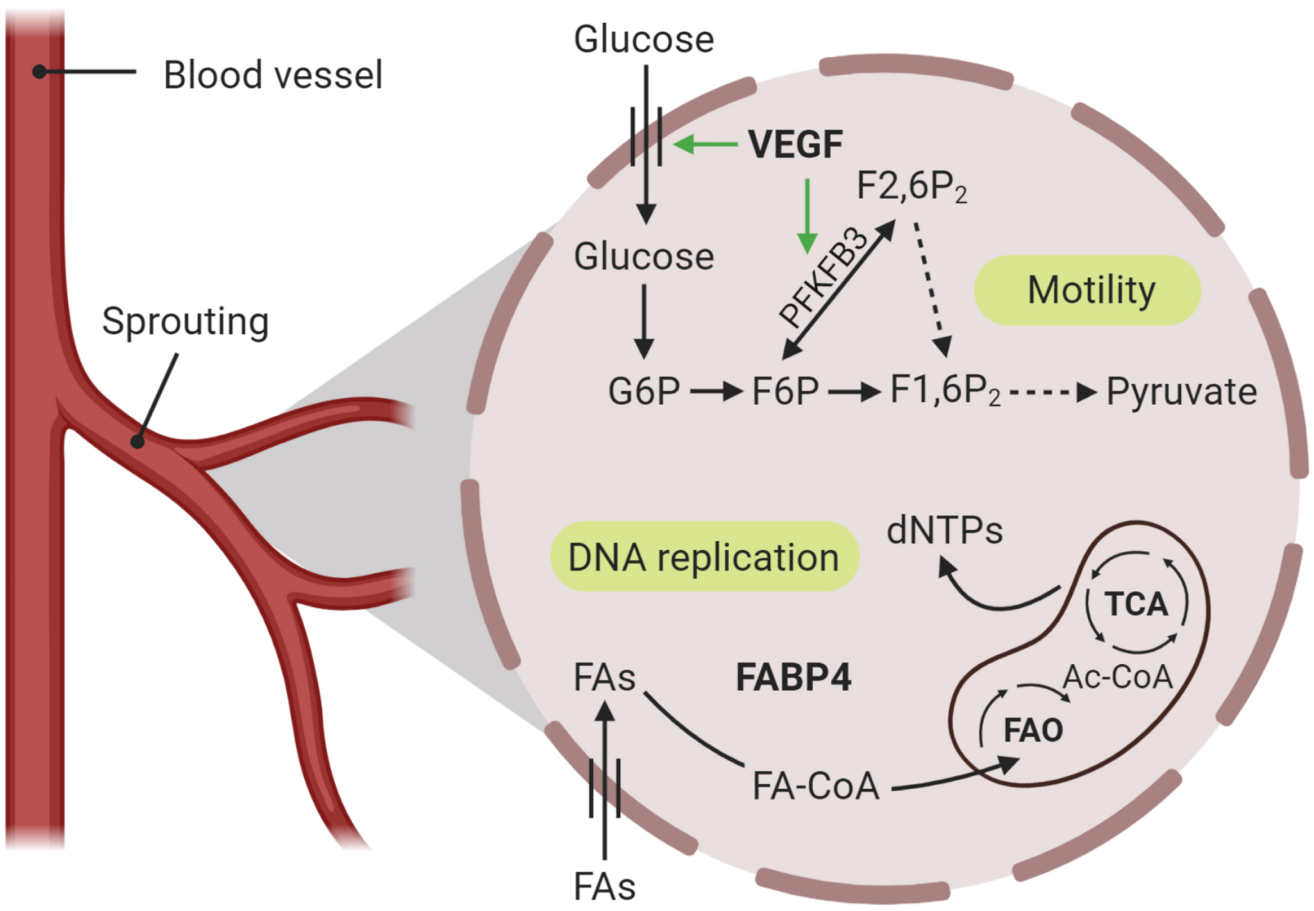
Endothelial cell metabolic pathways during vessel sprouting. Upon VEGF stimulation, the glycolytic flux is increased to meet the metabolic requirements of endothelial cells, enabling them to switch from quiescence to proliferation and vessel branching. VEGF induces the expression of the glucose transporters and up‐regulates the glycolytic enzyme 6‐phosphofructo‐2‐kinase/fructose‐2,6‐bisphosphatase 3 (PFKFB3), thus increasing proliferative and migratory capabilities of endothelial cells, which are necessary for vessel sprouting. Fatty acid oxidation (FAO) in endothelial cells is required for DNA replication. Fatty acids can enter the mitochondria to become oxidized and participate in the tricarboxylic acid (TCA) cycle, contributing to the de novo synthesis of nucleotides (deoxyribonucleotides [dNTPs]), necessary for endothelial cell proliferation during vessel sprouting. 6GP, glucose‐6‐phosphate; F1,6P2, fructose 1,6‐bisphosphate; F2,6P2, fructose 2,6‐bisphosphate; F6P, fructose‐6‐phosphate; FABP4, fatty acid‐binding protein 4; FAO, fatty acid oxidation; FAs, fatty acids

In spite of their access to high oxygen concentrations, ECs favour glycolysis over OXPHOS for their energy production, and they generate up to 85% of their ATP requirements through aerobic glycolysis. During inflammation and upon VEGF stimulation, the glycolytic flux is further increased to meet the metabolic requirements of ECs and thus enabling them to switch from quiescence to proliferation and vessel branching. VEGF induces the expression of GLUT1 by a mechanism involving the PI3K–Akt signalling pathway (Yeh, Lin, & Fu, 2008) and up‐regulates the glycolytic enzymes lactate dehydrogenase A **(**LDHA) and 6‐phosphofructo‐2‐kinase/fructose‐2,6‐bisphosphatase 3 (PFKFB3; De Bock, Georgiadou, Schoors, et al., 2013). PFKFB3 up‐regulates glycolysis via generation of fructose‐2,6‐bisphosphate, which is an allosteric activator of the rate‐limiting glycolytic enzyme phosphofructokinase‐1 (PFK1). Several studies have investigated the role of EC PFKFB3 up‐regulation in angiogenesis. For instance, PFKFB3 stimulates vessel sprouting in vitro and PFKFB3 silencing results in impairment of lamellipodia formation in ECs (De Bock, Georgiadou, Schoors, et al., 2013; Figure 3). A study by Schoors et al. (2014) has shown that blockade of PFKFB3 by 3‐(3‐pyridinyl)‐1‐(4‐pyridinyl)‐2‐propen‐1‐one (3PO) reduced vessel sprouting by inhibiting proliferative and migratory capabilities of ECs, thus reducing the pathological neovascularization in inflammatory models.

### 4.2 The pentose phosphate pathway

Besides the glycolytic pathway, glucose‐derived glucose‐6‐phosphate can be directed into the pentose phosphate pathway (PPP). The PPP is a cytoplasmic process, important for proliferating cells, consisting of a non‐oxidative branch that allows the diversion of intermediates from glycolysis towards the production of amino acids and nucleotides and an oxidative branch generating NADPH, a cofactor that supports lipid and cholesterol synthesis (Figure 2).

In contrast to healthy T cells, naïve T cells from RA patients show a defect in glycolytic flux due to the up‐regulation of glucose‐6‐phosphate dehydrogenase (G6PD). Excess G6PD shunts glucose into the PPP, resulting in NADPH accumulation and ROS consumption. Yang et al. (2016) have reported that this lack of ROS could boost pro‐inflammatory T cells in RA. The PPP is a key gatekeeper of inflammation, acting via the supply of ribose‐5‐phosphate for T‐cell proliferation. Additionally, it has been shown that the PPP enzyme transaldolase regulates susceptibility to Fas‐induced apoptosis by controlling the balance between mitochondrial ROS production and metabolic supply of reducing equivalents through the PPP (Banki, Hutter, Gonchoroff, & Perl, 1999). Alteration of this pathway and the resultant ATP depletion can sensitize T cells for necrosis, which may significantly contribute to inflammation in patients with systemic lupus erythematosus (SLE; Gergely et al., 2002).

For ECs too, there are several indications that the PPP plays a regulatory role in cell behaviour and angiogenesis. Upon activation of ECs during inflammation, VEGF induces the expression of GLUT1 resulting in increased intracellular glucose, which in turn up‐regulates not only the glycolytic enzymes but also the enzymes of the PPP. In ECs, G6PD regulates vascular tone through NO production and angiogenesis mediated by VEGF. It has been shown that G6PD translocates to the plasma membrane and is tyrosine phosphorylated by c‐Src at Y428 and Y507, which are required for VEGF‐induced Akt activation and EC migration (Pan, World, Kovacs, & Berk, 2009).

NADPH is a crucial cofactor for eNOS, which may explain the enhanced angiogenic properties in ECs overexpressing G6PD in response to VEGF. In response to VEGF stimulation, ECs overexpress G6PD, which in turn induces VEGFR2, Akt, and eNOS (tyrosine) phosphorylation, while G6PD silencing has the opposite effects, with a subsequent reduction in migration, proliferation, and tube formation by ECs, accompanied by increased cellular ROS production (Leopold, Zhang, Scribner, Stanton, & Loscalzo, 2003). Additionally, a study by Vizán et al. (2009) has shown that pharmacological inhibition of transketolase, the rate‐limiting enzyme in non‐oxPPP, limits EC viability and migration. To conclude, the PPP fuelled by glycolytic intermediates is crucial for EC metabolism during proliferation and angiogenesis, and hence, genetic or pharmacological inhibition of either branch of the PPP affects EC viability and migration as well as cellular ROS levels.

### 4.3 The TCA cycle

The TCA cycle is considered to be the central hub of cellular metabolism. Occurring in the matrix of the mitochondrion, it is responsible for the aerobic metabolism of any molecule that can be transformed into an acetyl group or dicarboxylic acid. The cycle also provides precursors necessary for the building of many molecules such as amino acids, nucleotide bases, cholesterol, and porphyrin. TCA cycle and OXPHOS are highly efficient modes of ATP generation. Two important products of this process are NADH and flavin adenine dinucleotide (FADH_2_), thus supporting oxidative phosphorylation (Figure 2).

As discussed above, there is a shift from the TCA cycle to glycolysis in activated T cells. However, the TCA cycle is very important for memory T cells, as they use the oxidation of endogenously produced FAs to produce acetyl‐CoA to support OXPHOS (O'Sullivan et al., 2014; van der Windt et al., 2013). FoxP3^+^ Tregs also use oxidative metabolism but rely on exogenously derived FAs to sustain this pathway (Gerriets et al., 2015; Michalek et al., 2011).

Glycolysis, FA oxidation, and glutaminolysis are the major carbon sources for TCA cycle intermediates, and several studies indicate that blockade of any of the enzymes controlling these pathways leads to a defective TCA cycle and consequently decreased proliferation of ECs during inflammation. Stone et al. (2018) have shown that loss of the enzyme PKM2 in ECs alters mitochondrial metabolism and the TCA cycle and impairs proliferation and migration of ECs by a mechanism involving NF‐κB transcription and p53 regulation. Activated ECs are distinguished from other highly proliferative cells such as cancer cells, by relying on FA‐derived acetyl‐CoA as the major carbon source for TCA cycle intermediates including citrate, α‐ketoglutarate, glutamate, and aspartate. Endothelial loss of the enzyme carnitine palmitoyl transferase (CPT1A) causes vascular sprouting defects due to impaired EC proliferation, linked to reduced deoxyribonucleotide synthesis (Schoors et al., 2015). Proliferating ECs also consume high amounts of glutamine and have high glutaminase (GLS1) activity, which forms glutamate, in a process known as glutaminolysis. Glutamate is then converted to α‐ketoglutarate, a TCA cycle intermediate, thus contributing to the enhanced protein and nucleotide synthesis during the proliferative state. GLS1 is up‐regulated in sprouting ECs in vivo, while glutamine depletion, or genetic inactivation of GLS1, dampens EC proliferation due to shortage of TCA metabolites, which results in defective macromolecular synthesis (Huang et al., 2017).

### 4.4 FA metabolism

FA metabolism includes FA synthesis and oxidation (Figure 2). The FA synthesis pathway produces lipids that are important for cellular proliferation and differentiation, starting from products derived from glycolysis, the TCA cycle, and the PPP. The FA oxidation pathway is responsible for the mitochondrial conversion of FAs leading to the final generation of acetyl‐CoA, NADH, and FADH_2_ that are further used in the TCA cycle and the electron transport chain to generate ATP.

It is well known that FAO is a key regulator of the balance between inflammatory effector T (Teff) cells and Tregs. Indeed, it has been reported that compared with Th1, Th2, and Th17 cells, which are highly glycolytic and down‐regulate FA oxidation, Tregs express low levels of GLUT1, use mitochondrial electron transport for proliferation, differentiation, and survival, and have high lipid oxidation rates (Michalek et al., 2011; Wang et al., 2011).

Memory CD8 T cells require FA oxidation to rapidly respond upon re‐stimulation, and this can explain why they show more mitochondrial mass than naïve CD8 cells. Moreover, IL‐15, a cytokine critical for memory CD8 T cells, promotes mitochondrial biogenesis and expression of CPT1A, the key metabolic enzyme of FA oxidation (van der Windt et al., 2013).

FA synthesis plays a critical role in the generation and function of pro‐inflammatory cells. It has been showed that de novo lipid synthesis is critical for the proliferation and function of Teff cells (Bensinger et al., 2008). The sterol regulatory element‐binding proteins (SREBPs) regulate FA and cholesterol synthesis in cells (Horton, Goldstein, & Brown, 2002) and genetic deletion of SCAP, an SREBP component, leads to significantly impaired growth and proliferation of T cells upon activation (Kidani et al., 2013). Additionally, PPARs, which regulate the lipid signalling pathway, have been shown to play a critical role in T‐cell activation, proliferation, and differentiation (Choi & Bothwell, 2012). FA synthesis is involved in the regulation of the balance between Teff cells and Tregs. Wang et al. (2015) showed that polyunsaturated FAs can reduce the expression of ligands for RORγT, a key Th17 transcription factor, thus reducing the expression of IL‐17 and increasing the expression of IL‐10. We have recently shown that ω‐3 polyunsaturated FAs can affect the motility of CD4 T cells and modify their ability to reach target tissues by interfering with the cytoskeletal rearrangements required for cell migration (Cucchi et al., 2019).

Emerging evidence indicates that the regulation of FA metabolism is also vital for EC metabolic reprogramming during angiogenesis and inflammatory response. FAs enter ECs via passive diffusion or by a specific FA translocase/CD36 (Harjes, Kalucka, & Carmeliet, 2016). FAs are believed to modify the lipid rafts of the EC membrane and hence facilitating TLR recruitment and activation (Goldberg & Bornfeldt, 2013). Moreover, saturated FAs bind to the free fatty acid (FFA1) receptor 1, a GPCR, thus modulating the EC inflammatory response via up‐regulation of IL‐6 (Lu et al., 2015). During inflammation and upon basic FGF and VEGF stimulation, the FA‐binding protein FABP4 can regulate proliferation and sprouting of ECs (Figure 3). Loss of FABP4 resulted in a significant decrease of EC proliferation, migration, and increased apoptosis, as well as impaired sprouting during angiogenesis (Elmasri et al., 2012). FAO plays a crucial role in angiogenesis. In fact, rather than using FAO for the production of energy or redox homeostasis, ECs use FAs for DNA synthesis (through the de novo synthesis of nucleotides, in particular deoxyribonucleotides), necessary for proliferation during vessel sprouting. This mechanism was confirmed in mice lacking CPT1A in ECs that show impaired sprouting (Schoors et al., 2015).

Apart from the supply of FAs from the blood stream, ECs can synthesize FAs, as they express the enzyme FA synthase (FASN). Acetyl‐CoA is used for FA production, and interestingly, through palmitoylation, FASN can regulate the bioavailability and membrane localization of eNOS, which generates NO and thus modulates the EC inflammatory response (Lu et al., 2015). FASN ablation not only impairs pathological angiogenesis and capillary formation but also attenuates palmitoylation and membrane localization of eNOS. This results in defective EC sprouting and increased EC permeability (Wei et al., 2011).

### 4.5 Amino acid metabolism

Amino acids, as the source of protein, are linked to the mTOR pathway and nucleotide synthesis. However, they can also be precursors for the de novo FA synthesis and de novo synthesis of purine and pyrimidine, and they can fuel the TCA cycle.

Amino acids are important substrates for T‐cell activation. Upon activation, T cells increase mRNA and protein expression of several glutamine transporters, including SLC38A1 and SLC38A2 (Carr et al., 2010). Glutamine can also be metabolized into glutamate and finally into TCA cycle intermediates, or used for FA synthesis via glutaminolysis, thus producing several other amino acids (alanine, aspartate, etc.), and can be a nitrogen donor for purine and pyrimidine nucleotide synthesis.

T‐cell receptor signalling regulates amino acid transporter expression and localization to the cell membrane (Nakaya et al., 2014; Sinclair et al., 2013; Figure 2). SLC7A5 is an amino acid transporter that regulates transport of large and branched neutral amino acids such as leucine and phenylalanine and is highly up‐regulated by antigen‐specific murine CD8 T cells during in vitro activation (Sinclair et al., 2013). Indeed, SLC7A5‐deficient murine CD4 and CD8 T cells are unable to proliferate, grow, and differentiate into Teff cells. The transporter SLC1A5, which regulates glutamine transport, is necessary for T‐cell proliferation, but its specific role is to modulate T‐cell differentiation and effector functions (Nakaya et al., 2014). SLC1A5 KO CD4 T cells exhibit impaired differentiation into Th1 and Th17 cells in vitro. These data suggest that different amino acid transporters have distinct regulatory effects on T‐cell activation.

Arginine metabolism also plays a crucial role in T‐cell function. Both in vitro and animal models have shown that the lack of arginine not only blocks T‐cell proliferation but also reduces the production of cytokines such as IFN‐γ, but not IL‐2 (Rodriguez et al., 2004).

In a recent study, it has been shown that extracellular alanine is required for efficient exit from quiescence during naive T‐cell activation and alanine depletion inhibits memory T‐cell re‐stimulation (Ron‐Harel et al., 2019).

As previously mentioned in Section 2.2, tryptophan and its downstream metabolites in the kynurenine pathway play a role in the modulation of T‐cell function. These cells need tryptophan to proliferate. T cells activated in the absence of free tryptophan enter the cell cycle, but cell cycle progression ceases in mid‐G_1_ phase and T cells become susceptible to death via apoptosis, in part through Fas‐mediated signalling (Lee et al., 2002).

Amino acids also have important regulatory effects on ECs. Glutamine significantly affects EC metabolism as it generates important metabolites for TCA cycle, nucleotide synthesis, and redox homeostasis and hence exerts several in vivo and in vitro angiogenic effects. Glutamine deficiency, genetic silencing, or pharmacological inhibition of GLS1 can impair EC proliferation (Huang et al., 2017). In vitro experiments have shown that both glutamine deprivation and inhibition of GLS1 prevent EC proliferation without affecting migration capabilities. The above‐mentioned effects were also accompanied by the complete loss of TCA cycle intermediates, which could not be compensated by glucose‐derived sources (Kim, Li, Jang, & Arany, 2017).

Glutamine‐derived non‐essential amino acids such as serine and glycine have also direct effects on ECs. In a recent study investigating the effects of glycine synthesis on EC metabolism during angiogenesis, oxidized phospholipids were involved in endothelial dysfunction via the induction of genes regulating serine–glycine metabolism (Hitzel et al., 2018). As discussed in Section 2.1, arginine can be metabolized by eNOS to citrulline and NO, a signalling molecule involved in the inflammatory immune response, maintenance of vascular homeostasis, modulation of T‐cell functions, and suppression of oxidative stress. On the other hand, branched‐chain amino acids (leucine, isoleucine, and valine) can markedly increase oxidative stress, NF‐κB activation, and inflammatory response in ECs (Zhenyukh et al., 2018). Methionine and cysteine also play an important role in EC metabolism. It has recently been proposed that sulfur amino acid restriction is a proangiogenic trigger, promoting increased VEGF expression, migration, and sprouting in ECs (Longchamp et al., 2018).

In this section, we have discussed the importance of metabolic pathways and substrates in the regulation of both EC and T‐cell activation and functions. A deeper understanding of the mechanisms underlying these functions is essential for the identification of pharmacological strategies aimed at perturbing or enhancing specific metabolic pathways involved in chronic inflammation‐related pathologies. We discuss this aspect in the next section of the review.

## 5 POTENTIAL FOR CLINICAL APPLICATIONS

**FIGURE 4:**
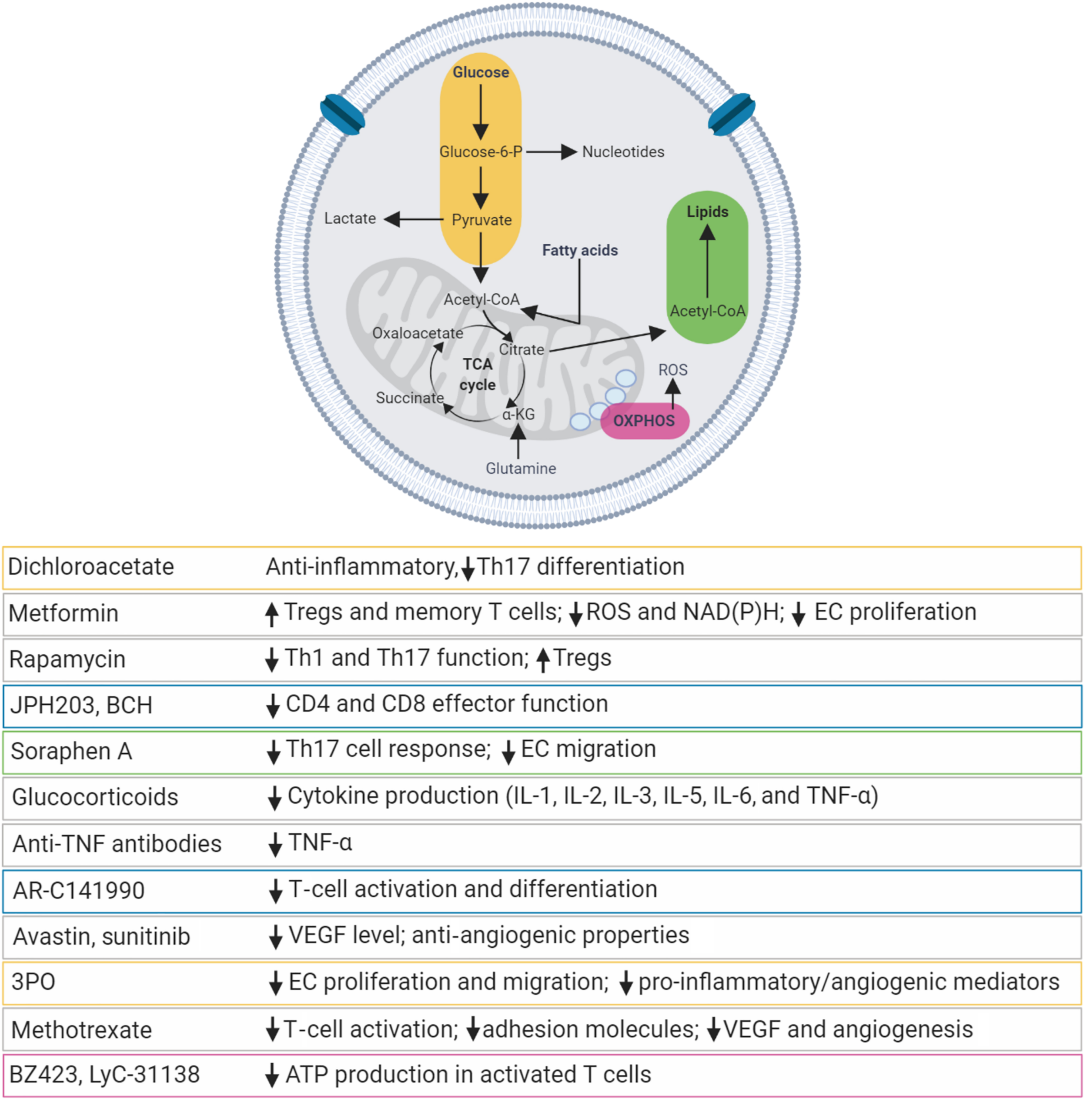
Therapeutic approaches targeting endothelial cell (EC) and T‐cell metabolism. EC and T‐cell activation requires metabolic reprogramming to cope with increased energy demand. The diagram highlights some of the most important metabolic pathways involved in this metabolic switch. The table indicates some examples of potential therapeutic strategies that may be used to target EC and T‐cell metabolism, in order to mitigate the chronic inflammatory response. OXPHOS, oxidative phosphorylation; TCA, tricarboxylic acid; α‐KG, α‐ketoglutarate

Several studies have attempted to manipulate EC and T‐cell functions through interventions directed at metabolic pathways, highlighting potential therapeutic strategies in the context of chronic inflammation (Figure 4). Although there are numerous studies on EC–T‐cell interactions, little is yet known on their reciprocal influences in terms of metabolic reprogramming. However, drugs that modulate the metabolism of one cell type certainly can have repercussions on the other type. Of greater importance is the identification of drugs that can modulate the metabolism and, therefore, the activity of both cell types simultaneously.

Methotrexate is an antimetabolite of the antifolate family and is used in the treatment of autoimmune diseases, including RA. It acts via the inhibition of enzymes involved in purine metabolism, leading to accumulation of adenosine, and via the suppression of T‐cell activation and expression of adhesion molecules (Yamasaki, Soma, Kawa, & Mizoguchi, 2003). However, methotrexate can also reduce VEGF expression and neovascularization (Shaker et al., 2013). This can be due to a direct effect or to an indirect effect via the reduced expression of cytokines, including IL‐1 and TNF‐α, which are potent angiogenic factors.

Metformin is the first‐line therapy for Type 2 diabetes mellitus and is now receiving increasing attention due to its anti‐inflammatory properties. In addition, it has been reported that metformin has protective effect on ECs in diabetes and atherosclerosis, through the reduction of oxidative stress, inhibition of NAD(P)H oxidase, and activation of eNOS (Batchuluun et al., 2014; Valente, Irimpen, Siebenlist, & Chandrasekar, 2014). In some clinical conditions, metformin can also exert anti‐proliferative effects on ECs. Therefore, this is another example, along with that of methotrexate, of a drug capable of exerting beneficial effects on both ECs and T cells in the context of chronic inflammatory diseases.

The development of specific drugs that simultaneously target EC and T‐cell function could lead to the generation of more valuable therapeutics.

### 5.1 Targeting T‐cell metabolism

It has been suggested that targeting PDHK1, an enzyme that inhibits pyruvate dehydrogenase, with dichloroacetate, blocks Th17 differentiation and cytokine production while leaving Th1 unaffected. This may be important in the context of Th17‐driven autoimmune disorders, where it would favour the suppression of the Th17 lineage without affecting Th1 immunity (Gerriets et al., 2015). Dichloroacetate treatment was also able to reduce lactate production and alleviate inflammation (Ostroukhova et al., 2012).

Inhibitors of the F1/F0 ATPase, such as BZ423 and LyC‐31138, have demonstrated promise as a metabolic therapy for graft‐versus‐host disease (GVHD) by preventing the production of ATP in pathogenically activated lymphocytes (Glick et al., 2014). The same kind of inhibition has been shown to promote apoptotic death of chronically stimulated T cells in models of SLE (Gatza et al., 2011). These effects can be explained by the fact that the generation of ATP in chronically stimulated cells from autoimmune models and in GVHD is mainly derived from oxidative metabolism.

AMP‐activated protein kinase (AMPK) was reported recently to have anti‐inflammatory activities by negatively regulating NF‐κB signalling. By using the experimental autoimmune encephalomyelitis model, Nath et al. (2005) showed that the activator of AMPK, 5‐aminoimidazole‐4‐carboxamide ribonucleoside (AICAR), attenuated the severity of the clinical disease through the inhibition of Th1‐type cytokines, IFN‐γ and TNF‐α, and the production of Th2 cytokines, IL‐4 and IL‐10.

Similar results have been described in bowel disease, which is characterized by excessive Th1 and Th17 immune responses (Bai et al., 2010). As mentioned above, metformin can directly modulate the functions of various immune cell types and suppress the immune response mainly through indirect activation of AMP and subsequent inhibition of mTORC1 and by inhibition of mitochondrial ROS production. In addition, metformin treatment increases differentiation of T cells into both Tregs and memory T cells (Sun et al., 2016). Yin et al. (2015) reported that CD4 T cells isolated from SLE patients displayed increased glycolysis and OXPHOS. Treatment with a combination of metformin and 2‐deoxyglucose was able to normalize T‐cell metabolism, and therefore, it represents a promising therapeutic approach for the treatment of SLE.

Rapamycin is an mTOR inhibitor that selectively inhibits Th1 and Th17 proliferation (Delgoffe et al., 2011). At the same time, it has been shown that rapamycin promotes Treg survival and function leading to an enrichment of Tregs in mice and humans (Battaglia, Stabilini, & Roncarolo, 2005), proving to be useful in multiple sclerosis and SLE (Perl, 2015).

Another potential approach to target T‐cell metabolism for therapy is to limit the availability of nutrients. T‐cell‐specific deletion of GLUT1 impairs CD4 T‐cell activation, expansion, and survival (Macintyre et al., 2014). Importantly, GLUT1 deficiency was able to reduce Teff cell expansion and the inflammatory response without affecting Treg cell functions. CD8 T cells are less functional following glucose deprivation, and they show reduced IFN‐γ, granzyme, and perforin production (Cham, Driessens, O'Keefe, & Gajewski, 2008).

Deletion of the neutral amino acid transporter SLC7A5, which is important in coordinating the metabolic reprogramming essential for Th1 and Th17 cell differentiation and function, or its inhibition with JPH203 and 2‐aminobicyclo‐(2,2,1)‐heptane‐2‐carboxylic acid (BCH), prevents the metabolic reprogramming, expansion, and/or effector function of both CD4 and CD8 T cells, without affecting Treg cell differentiation (Sinclair et al., 2013). During T‐cell activation, there is also an increased expression of the alanine serine and cysteine transporter system (ASCT2/SLC1A5). The loss of ASCT2 leads to the impaired polarization of T cells towards a Th1/Th17 phenotype but not Th2 or Treg (Nakaya et al., 2014).

Numerous studies in recent years have revealed the special importance of cellular metabolism of FAs in T‐cell differentiation. Indeed, while de novo FA synthesis is crucial for proliferation and differentiation of Teff cells, β‐oxidation of FA is important for the development of CD8 T‐cell memory as well as for the differentiation of CD4 Tregs (Lochner, Berod, & Sparwasser, 2015). A reduced expression of SREBP1 and SREBP2 protein, crucial transcription factors in lipid metabolism, impairs T‐cell activation and proliferation (Yang et al., 2013). Also, in vivo treatment with the ACC‐specific inhibitor soraphen A or T‐cell‐specific deletion of ACC1 in mice impairs the Th17 cell response in autoimmunity (Berod et al., 2014). ACC1 deficiency also impairs Th1 and Th2 development, suggesting that FA synthesis is fundamental for CD4 effector T‐cell functions (Berod et al., 2014).

Alloreactive Teff cells use FAs as a fuel source to support their in vivo activation, and pharmacological blockade of FA oxidation is able to decrease the survival of alloreactive T cells without influencing the survival of T cells during normal immune reconstitution (Byersdorfer et al., 2013). These studies suggest that modulation of FA metabolism with drugs such as etomoxir, can be a useful strategy for the treatment of GVHD and other T‐cell mediated immune diseases, which present high rates of FAO.

There is emerging evidence that metabolic enzymes and regulators can have a direct role in controlling immune cell functions, thus proving to be potential therapeutic targets. It has been reported that inhibiting the glycolytic enzyme hexokinase‐2 with 3‐bromopyruvate can polarize Th17 cells towards Tregs after their stimulation in vitro and, thus, ameliorate the inflammatory response in a mouse model of experimental arthritis (Okano et al., 2017).

Recent studies have revealed an important role for glycolysis and the glycolytic enzyme enolase in controlling the induction and suppressive function of iTregs. This is mainly due to the control of Foxp3 splicing variants containing exon 2 (Foxp3–E2) through the glycolytic enzyme enolase‐1. When glycolysis is reduced, enolase can translocate to the nucleus and inhibit the formation of Foxp3–E2 iTregs (De Rosa et al., 2015). This suggests that targeting enolase nuclear translocation may be able to sustain the formation of these immunosuppressive Foxp3–E2‐expressing iTregs.

LDHA is the enzyme responsible for the conversion of pyruvate to lactic acid and deletion of LDHA impaired Th1 differentiation and function. This was a consequence of the shunting of acetyl‐CoA into the TCA cycle, resulting in a reduced amount of acetate available for epigenetic regulation (Peng et al., 2016). These results suggest that aerobic glycolysis, via epigenetic mechanisms, can promote Teff cell differentiation and suggest that LDHA may be a therapeutic target in inflammatory diseases.

The glycolytic enzyme pyruvate kinase functions as a homo‐tetramer in the cytosol converting phosphoenolpyruvate to pyruvate in the last reaction of glycolysis. PKM2 is the major isoform expressed at the protein level by lymphocytes (Dayton et al., 2016). Dimers of PKM2 localize in the nucleus where they can modulate inflammatory programs by interacting, for instance, with STAT3 (Gao, Wang, Yang, Liu, & Liu, 2012) and the aryl hydrocarbon receptor (AhR; Matsuda et al., 2016). Thus, enforcing PKM2 tetramerization can be a useful strategy for the attenuation of inflammatory responses, and several studies have reported the achievement of these results in different diseases.

It has been reported that AhR activation increases FoxP3^+^ Tregs through different mechanisms, including direct transactivation and the induction of epigenetic modifications that control Foxp3 transcription (Goettel et al., 2016). Kynurenine, a metabolite derived from IDO‐mediated tryptophan catabolism, is a suppressor of CD8 T‐cell proliferation and effector function (Munn & Mellor, 2013). It can lead to increased production of anti‐inflammatory Tregs and decreased pro‐inflammatory Th17 expansion via an AhR‐dependent mechanism, and this evidence further consolidates the importance of this signalling pathway in the treatment of inflammatory and autoimmune diseases (Busbee, Rouse, Nagarkatti, & Nagarkatti, 2013).

Different levels of metabolites can also lead to modifications of cellular functions. For instance, ROS has emerged as an important second messenger for T‐cell receptor signalling, T‐cell activation, and proliferative expansion (Yarosz & Chang, 2018). It has been reported that activation of the nuclear factor erythroid 2‐related factor 2‐mediated antioxidant pathway in T cells reduces inflammation in the experimental autoimmune encephalomyelitis (Kuo et al., 2016). Several studies have also shown that compounds like triterpenoids, 3*H*‐1,2‐dithiole‐3‐thione (D3T), and cannabidiol can activate nuclear factor erythroid 2‐related factor 2 in T cells, generating anti‐inflammatory effects by decreasing differentiation or cytokine production in Th1 and Th17 cells (Kozela et al., 2016; Kuo et al., 2016).

In terms of cytokine production, glucocorticoids such as prednisone, dexamethasone, and hydrocortisone are commonly used to counteract inflammatory and autoimmune disorders. They are also used to prevent transplant rejection and GVHD. These drugs inhibit the production of IL‐1, IL‐2, IL‐3, IL‐5, IL‐6, and TNF‐α, although these compounds can induce resistance in certain circumstances (Newton, Shah, Altonsy, & Gerber, 2017). TNF‐α is linked to a wide variety of autoimmune diseases, including RA, psoriasis, SLE, and diabetes. Systemic inhibitors of TNF such as etanercept (a soluble TNF receptor), infliximab and adalimumab (anti‐TNF antibodies) have been approved for the treatment of psoriasis and RA (Meier, Frerix, Hermann, & Muller‐Ladner, 2013). Golimumab and certolizumab pegol are two TNF inhibitors with higher efficiency in the treatment of patients with RA (Li et al., 2017).

As previously highlighted, lactate plays an important role in the regulation of immune cell functions. We have recently reported that pharmacological targeting of the lactate transporter SLC5A12 shows promising results in a preclinical model of RA characterized by the infiltration of CD4 T cells (Pucino et al., 2019). Also, inhibition of MCT1 during T‐lymphocyte activation results in selective and profound inhibition of the extremely rapid phase of T‐cell division, essential for an effective immune response (Murray et al., 2005). Compounds, such as AR‐C141990, that block MCT‐1 are efficacious in blocking alloimmune responses, such as the graft‐versus‐host response, and in preventing cardiac allograft rejection (Påhlman et al., 2013). Based on this evidence, targeting lactate transporters may become a promising therapeutic avenue for the management of chronic inflammatory diseases.

### 5.2 Targeting EC metabolism

Emerging studies are highlighting the role of metabolic pathways in regulating pathological angiogenesis and targeting EC metabolism is starting to be considered a new potential therapeutic strategy in the context of chronic inflammatory diseases.

The important role of glycolysis in angiogenesis, for example, can provide opportunities for therapeutic targeting. Indeed, blockade of PFKFB3 by 3PO reduced vessel sprouting by inhibiting EC proliferation and migration (Schoors et al., 2014). Another study has shown that inhibition of PFKFB3 reduced the secretion of pro‐inflammatory/angiogenic mediators in RA–fibroblast‐like synoviocytes and ECs, thus further suggesting a key role of this glycolytic enzyme in promoting angiogenesis (Biniecka et al., 2016).

Given the important role of VEGF in the process of RA and synovitis, antagonizing VEGF could be an efficient strategy in the treatment of these conditions. In this context, it has been reported that treatment with bevacizumab, a humanized monoclonal antibody against VEGF, decreased the serum VEGF levels and arthritis index (Wang, Da, Li, & Zheng, 2013). In another study, sunitinib, an angiogenesis inhibitor that targets tyrosine kinases of the VEGFR family, decreases the disease score in a murine model of arthritis (Furuya, Kaku, Yoshida, Joh, & Kurosaka, 2014). Therefore, there is considerable evidence suggesting how inhibitory molecules aimed at blocking VEGF signalling can be beneficial in inflammatory diseases.

Some studies have shown that lactate promotes angiogenesis in vivo. Indeed, lactate can enter ECs through MCT1 and induce HIF‐1α with subsequent increased expression of VEGFR2 and basic FGF. MCT1 inhibition can exert direct anti‐angiogenic effects through a reduction in HIF‐1 (Sonveaux et al., 2012). Inhibition of MCT1 can also reduce lactate‐induced NF‐κB activation in ECs, and this leads to a reduced IL‐8 signalling and IL‐8‐mediated angiogenesis (Vegran, Boidot, Michiels, Sonveaux, & Feron, 2011).

In the context of chronic inflammation, there are several studies indicating eNOS activity as a fundamental target. Indeed, eNOS dysfunction is the primary cause of ROS production and EC damage (Mugoni et al., 2013). It is well known that NO has a protective effect at low concentrations, through the induction of a population of Tregs, which could counteract a potentially damaging autoimmune response (Niedbala et al., 2006). However, it has been reported that iNOS‐derived overproduction of NO can lead to an increased production of pro‐inflammatory mediators, through the activation of NF‐κB. Based on these observations, it has been suggested that CoQ10 might have a positive role in modulating NO‐related pathways by recoupling eNOS in ECs, and its beneficial effects are mainly due to the suppression of NF‐κB and NF‐κB‐related pro‐inflammatory response (Tsai et al., 2012).

Several studies have shown that ω‐3 fatty acids have an anti‐angiogenic effect, in addition to their anti‐inflammatory activity. These effects on ECs are mainly associated with reduced expression of VEGFR2, MMP‐2, and MMP‐9 (Tsuzuki, Shibata, Kawakami, Nakagawa, & Miyazawa, 2007).

Li et al. have shown that intracellular succinate can regulate angiogenesis via HIF‐1α induction, while extracellular succinate can modulate the activation of the succinate receptor GPR91, thus altering energy metabolism and exacerbating inflammation and angiogenesis in arthritis. Suppression of succinate dehydrogenase could prevent succinate accumulation and inhibit angiogenesis via blocking HIF‐1α/VEGF axis (Li et al., 2018). This finding reveals a potential therapeutic strategy to attenuate revascularization in chronic inflammatory diseases and suggests that succinate can act as a signalling molecule to link metabolic reprograming with angiogenesis.

Finally, depriving ECs of glutamine or inhibiting GLS1 can reduce vessel sprouting leading to impaired proliferation and migration as a consequence of impaired TCA cycle anaplerosis, macromolecule production, and redox homeostasis (Huang et al., 2017).

## 6 CONCLUSIONS

In recent years, metabolic reprogramming has been increasingly considered as a therapeutic modality, as it is becoming clear that it plays a crucial role in the coordination of the immune response. During inflammation, several drastic changes have been described in both ECs and T cells. ECs play important roles in T‐cell‐mediated immune functions and can modulate metabolic reprogramming of T cells through secretion of various signalling molecules. Similarly, several studies indicate that T cells play significant roles as regulators of EC functions during inflammation through secretion of many immunomodulatory molecules and cytokines. This review provides a comparison between these two systems and highlights how they affect each other. At the same time, the main metabolic pathways induced during the activation of ECs and T cells are described. Understanding the mechanisms that drive metabolic reprogramming in these cells can lead to the explanation of the pathogenesis of numerous diseases, and new therapies can be developed based on the fact that many metabolic enzymes can be pharmacological targets. This review illustrates some examples of targeting EC and T‐cell metabolism highlighting promising therapeutic interventions for the selective regulation of EC and T‐cell functions in the context of chronic inflammatory disorders.

### 6.1 Nomenclature of targets and ligands

Key protein targets and ligands in this article are hyperlinked to corresponding entries in http://www.guidetopharmacology.org, the common portal for data from the IUPHAR/BPS Guide to PHARMACOLOGY (Harding et al., 2018), and are permanently archived in the Concise Guide to PHARMACOLOGY 2019/20 (Alexander, Cidlowski et al., 2019; Alexander, Fabbro et al., 2019a, 2019b; Alexander, Kelly et al., 2019a, 2019b).

## CONFLICT OF INTEREST

The authors declare no conflicts of interest.
